# Platelet Membrane–Encapsulated MSNs Loaded with SS31 Peptide Alleviate Myocardial Ischemia-Reperfusion Injury

**DOI:** 10.3390/jfb13040181

**Published:** 2022-10-09

**Authors:** Zaiyuan Zhang, Zhong Chen, Ling Yang, Jian Zhang, Yubo Li, Chengming Li, Rui Wang, Xue Wang, Shuo Huang, Yonghe Hu, Jianyou Shi, Wenjing Xiao

**Affiliations:** 1 College of Medicine, Southwest Jiaotong University, Chengdu 610031, China; 2 Department of Ultrasound, The General Hospital of Western Theater Command of PLA, Chengdu 610083, China; 3 School of Clinical Medicine, Chengdu University of TCM, Chengdu 610072, China; 4 College of Integrated Traditional Chinese and Western Medicine, Southwest Medical University, Luzhou 646000, China; 5 Personalized Drug Therapy Key Laboratory of Sichuan Province, Department of Pharmacy, Sichuan Academy of Medical Sciences & Sichuan Provincial People’s Hospital, School of Medicine, University of Electronic Science and Technology of China, Chengdu 610072, China; 6 Department of Pharmacy, The General Hospital of Western Theater Command of PLA, Chengdu 610083, China; 7 School of Materials Science and Engineering, Southwest Jiaotong University, Chengdu 611756, China

**Keywords:** ischemia-reperfusion, oxidative damage, mitochondria-mediated apoptosis, drug delivery, platelet membrane

## Abstract

Clinically, antioxidant therapy is a potential strategy for myocardial ischemia-reperfusion injury (MI/RI), a common complication of acute myocardial ischemia. The H-D-Arg-Dmt-Ly-Phe-NH_2_ (SS31) peptide is shown to have amazing antioxidant properties, but its utilization is limited by the peptide characteristics, such as the destruction by proteases and rapid metabolism. Silica nanoparticles (MSNs) comprise an excellent material for peptide delivery, owing to the protection effect relating to peptides. Moreover, platelet membrane (PLTM) is shown to be advantageous as a coat for nanosystems because of its specific protein composition, such that a PLTM-coated nanosystem has a stealth effect in vivo, able to target injury in the cardiovascular system. Based on this feature, we designed and prepared a novel nanocarrier to target SS31 delivery. This carrier is encapsulated by a platelet membrane and loaded with SS31 peptide into MSNs. The results reveal that this delivery system can target SS31 to the injured cardiovascular site, exert antioxidant function, and alleviate MI/RI.

## 1. Introduction

Cardiovascular disease is extremely harmful; it is a category of diseases that needs to be addressed urgently in the clinic [1]. In particular, myocardial ischemia-reperfusion injury (MI/RI) is a typical complication in the treatment of acute myocardial ischemia [2,3]. 

Among the mechanisms discovered so far, oxidative stress injury is a common cause of MI/RI. In the first few seconds of reperfusion, a large number of free radicals are generated in the myocardium. These free radicals are key substances in the process but are considered to be the main cause of reperfusion injury [4]. The produced superoxide anions and their by-products attack cardiomyocytes and co-stimulate myocardial tissue with other types of reactive oxygen species (ROS), triggering oxidative stress [5]. The increase of oxygen free radicals leads to the relative insufficiency of the corresponding scavenging system, reducing the scavenging of these radicals and converting them into strong oxidative hydroxyl radicals (HO·). This process promotes the opening of mPTP, which causes mitochondria to swell or even rupture from changes in osmotic pressure. The result is dysfunction and further increases in the ROS level, forming a cascade relationship and causing cell necrosis and apoptosis [6]. Therefore, the increased level of ROS is a key factor in MI/RI [7]. 

**Figure 1:**
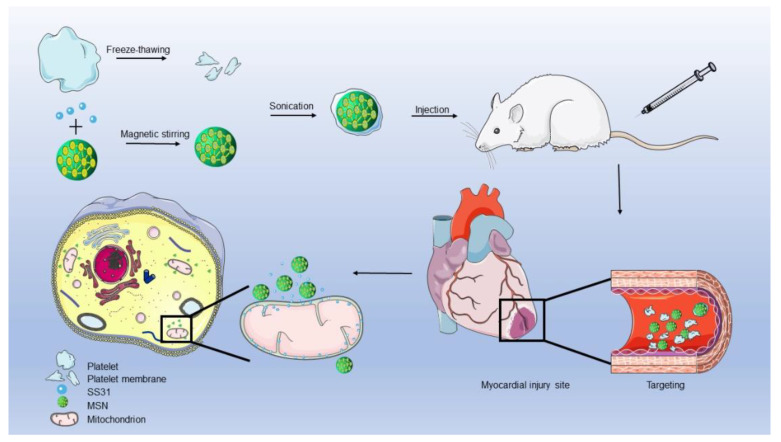
Schematic diagram of material synthesis and its effect in rats.

In this paper, a targeted drug delivery system was designed, as shown in Figure 1. SS31 peptide is loaded into silica nanoparticles (MSNs) and wrapped by a platelet membrane (PLTM), or SS31/MSN@PLTM. Previous studies have shown that SS31 has antioxidant properties with the ability to reduce intracellular ROS levels in pathological conditions, similar to the function of natural SOD enzymes [8,9]. Antioxidants are one of the key modes for myocardial ischemia-reperfusion support, but the ability of SS31 to alleviate myocardial ischemia-reperfusion is not evident, probably due to its poor stability under physiological conditions. SS31 utilization in the clinic is limited by its peptide characteristics, such as the destruction by proteases [10]. As a peptide, SS31 may be destroyed by proteases, and in order to overcome this limitation, this synthetic peptide was firstly encapsulated into MSNs; these are potential nanocarriers for peptides, owing to their stable release drug characteristics [11,12,13,14,15,16]. MSNs were then coated by PLTM to avoid their being swallowed by macrophages in the body [17,18]. As a natural component of blood, platelets can also target sites of cardiovascular injury thanks to specific proteins on their membranes and their adhesive properties [19,20,21]. In this study, the SS31 peptide was used for the treatment of myocardial ischemia-reperfusion in rats for the first time. According to the characteristics of the peptide, we designed a specific targeted drug delivery system, which provides a potential therapeutic method for the treatment of MI/RI. 

## 2. Materials and Methods

### 2.1. Materials

The following materials were used in this study:

- MSN (purchased from Merck, Kenyos, NJ, USA)
- SS31 (purchased from Nanjing Jill Chemical Co., Ltd., Nanjing, China)
- DMEM medium (purchased from Hyclone, Utah Logan, UT, USA)
- Trypsin
- Super grade fetal bovine serum
- DMEM medium (purchased from GIBCO, Karlsbad, CA, USA)
- CCK8 enhanced reagent
- Reactive oxygen species detection kit
- Enhanced JC-1 detection kit
- Evans blue, 2, 3, 5-triphenyl tetrazole chloride (TTC)
- Recombinant anti-Bax antibody
- Recombinant anti–cytochrome-C antibody
- Recombinant anti–caspase-3 antibody, rabbit anti-mouse APAF antibody
- Rabbit anti-rat Bcl-2 antibody
- Rabbit anti-rat caspase-9 antibody
- Malondialdehyde kit
- Rat SOD ELISA kit
- Rat CK-MB ELISA kit
- Rat cTnT ELISA Kit (purchased from Beyotime Biotechnology, Shanghai, China)
- PGE1(purchased from Roche, Basel, Switzerland)
- Male Sprague-Dawley (SD) rats (purchased from Chengdu Dossy Experimental Animals Co., Ltd., Chengdu, China)
- Rat cardiomyoblast-derived cells (H9c2; purchased from Procell Life Science & Technology Co., Ltd., Wuhan, China))

### 2.2. Methods

#### 2.2.1. PLTM Preparation

To prepare the PLTM, we collected whole rat blood. Rat blood was collected through the abdominal aorta and PLTM was obtained according to previous studies [17,22]. Whole blood was centrifuged at 100 g for 20 min at room temperature and erythrocytes were discarded to obtain platelet rich plasma (PRP). PRP was centrifuged at 100 g for 20 min. PBS buffer containing 1 mM EDTA and 2 mM prostaglandin E1 (PGE1, Roche) was added to the purified PRP, and then centrifuged at 800 g for 20 min at room temperature; the supernatant was discarded and resuspended in PBS containing 1 mM EDTA and protease inhibitors to obtain a platelet suspension. The suspension was frozen at −80 °C and thawed at room temperature. This was repeated three times, and followed by centrifugation at 4000 g for 3 min at room temperature to obtain a platelet suspension. The platelets were washed for three times and dispersed in 1.5 mL of DEPC water. Finally, PLTM suspension was obtained by sonication at 40 kHz and 100 W for 5 min at room temperature. 

#### 2.2.2. Synthesis of SS31/MSN@PLTM

To synthesize SS31/MSN@PLTM, 1 mg of MSNs was dispersed in DEPC water, 3 mg of SS31 peptide was added, and magnetic stirring was carried out in an ice bath for 3 h. After stirring, 1.5 mL PLTM suspension (2 × 10^9^/mL) was added and ultrasonicated for 2 min (FS30D bath sonicator). Finally, SS31/MSN@PLTM was re-suspended in 300 μL DEPC water [17,23].

#### 2.2.3. SS31/MSN@PLTM Characterization

The stability of SS31/MSN@PLTM was tested by Malvern Zetasizer Nano ZS. 

The morphology and structure of the materials were characterized by a scanning electron microscope (SEM; FEI Apreo S) and transmission electron microscope (TEM; JEM-1400 FLASH). In addition, to detect whether PLTM was successfully wrapped, SDS gel electrophoresis was performed, using the following procedure. Take 15 uL sample and 5 uL 4× loading buffer and treat it at 95 °C for three minutes. Carefully add the prepared samples into the loading tank, and add 5 uL marker at the appropriate position. Set the running parameters as current max and voltage 100 V, and stop running when the protein is close to the bottom of the gel. Wash the residual running buffer with ddH_2_O, then immerse the gel in Coomassie brilliant blue staining solution for 2 h and treated it with destaining solution (30% methanol, 10% acetic acid, 60% ddH_2_O) until the protein bands are clearly visible. Recorded the data by taking pictures with a Bio-Rad recorder. 

We studied the release behavior of SS31 by dialysis. We placed the SS31/MSN@PLTMs in a dialysis bag (molecular weight cut-off 8000–14000), adding 1 mL of ultrapure water; both ends were sealed and then placed in PBS with pH 7.4. We measured the solution at 30 min, 1 h, 2 h, 4 h, 8 h, and 16 h. Each time, the same amount of PBS was added and filtered, and the concentration of SS31 in the supernatant was calculated using Pierce Quantitative Fluorometric Peptide Assay. Quantitative detection of SS31 was performed according to the instructions. 10 µL of the prepared standard or sample was pipetted into a fluorescence-compatible microplate. 70 µL of Fluorescent Peptide Detection Buffer was added to each well and 20 µL of Fluorescent Peptide Detection Reagent to each well, followed by incubation at room temperature for 5 min. Fluorescence was measured at 390 nm/475 nm using Ex/Em. The peptide concentration of each sample was determined from the standard curve. The release curve was then drawn. 

#### 2.2.4. Cell Culture

After the H9c2 cells were cultured in the conventional medium for 24 h, the cell morphology was observed. If the cells were in good condition and in the logarithmic growth phase, the medium was replaced with serum-free DMEM low glucose medium (50 mL low glucose medium containing 49.5 mL DMEM low glucose medium and 0. 5 mL penicillin-streptomycin solution) and placed in the hypoxia incubator (37 °C, 5% carbon dioxide, 95% nitrogen) for 8 h. After hypoxia, we discarded the low-sugar medium, added a regular medium, and reoxygenated it in a conventional incubator for 2 h (37 °C, 5% carbon dioxide, 95% air). After reoxygenation, the hypoxia-reoxygenation (H/R) injury cell model was established. The cell seeding density of the 96-well plate was 5 × 10^4^ cells/mL, and the 6-well plate was 3 × 10^5^ cells/mL. 

RAW 264. 7 (macrophage-like) cells were cultured in high glucose DMEM medium with 10% FBS. 

#### 2.2.5. Toxicity Studies on SS31/MSN@PLTM

The cytotoxicity of SS31/MSN and SS31/MSN@PLTM was tested on H9c2 cells using the CCK8 assay. We treated the cells with a range of concentrations of the material suspension and incubated them for 24 h. After the incubation period, we added 10 μL of CCK8 solution to each well and incubated these for 30 min. After incubation, we measured the absorbance at 450 nm using a plate reader to analyze the result (BioTek Instruments, Winooski, VT, USA). 

#### 2.2.6. Mitochondrial Localization

To evaluate the mitochondrion-localizing properties of these newly synthesized SS31/MSN@PLTMs, FITC-labeled SS31 [24] was loaded into the MSNs and encapsulated with PLTMs and red cell membranes (RBM), respectively. We labeled the cell mitochondria with Mito-Red and the cell nuclei with Hoechst, and the three groups of nanomaterials were each incubated with H/R H9c2 cells. After incubation, fluorescent staining was observed using an Olympus automatic inverted microscope, and the Pearson correlation coefficient [25] of each group was calculated to analyze the mitochondrial localization level of each group. 

#### 2.2.7. Measurement of Intracellular ROS

We used a reactive oxygen species detection kit to analyze the level of ROS. First, we stained the cells with 2, 7-dichlorodihydrofluorescein diacetate (DCFH-DA) [26] according to the manufacturer’s instructions after different treatments. Then, the green fluorescence of cells in each group was observed using a fluorescence microscope. 

#### 2.2.8. Detection of Change in Mitochondria Membrane Potential

We used the fluorescence microscope to detect the 5, 5′, 6, 6′-tetrachloro-1, 1′, 3, 3′-. tetraethylbenzi-midazolylcarbocyanine iodide (JC-1) staining [27]. After incubation with the JC-1 staining solution at 37 °C in the cell incubator for 10 min, cells were washed with JC-1 staining buffer two times and then observed. 

#### 2.2.9. Transmission Electron Microscopy 

The samples were pre-fixed with 3% glutaraldehyde, re-fixed with 1% osmium tetroxide, dehydrated in acetone, and embedded in Ep812. Next, semi-thin sections were stained with toluidine blue for optical positioning, and ultra-thin sections were made with a diamond knife, uranyl acetate, and lead citrate staining [28]. We observed the process using a JEM-1400FLASH TEM. 

#### 2.2.10. Flow Cytometry

Apoptotic H9c2 cardiomyocytes were measured using flow cytometry. Each group of cell culture medium was carefully collected in a centrifuge tube and set aside. The cells digested with trypsin were added to the previous centrifuge tube, centrifuged at 1000 g for 3 min, and the supernatant was discarded. Cells were resuspended in 1 mL of ice PBS, washed, and resuspended again. Cells were first stained with Annexin V-FITC for 10 min and propidium iodide (PI) for 5 min (Beijing 4A Biotech Co., Ltd, Beijing, China) at room temperature [29]. Immediately after the incubation, flow detection was performed, and the results were analyzed using CytExpert software (Beckman Coulter, Inc., Brea, CA, USA). 

#### 2.2.11. Establishment of the Rat Myocardial Ischemia-Reperfusion Model

The rats were each anesthetized with an intraperitoneal injection of 1% pentobarbital solution and tracheal intubation and connected to a small animal ventilator [30]. At the same time, an electrocardiograph was used to monitor the rats’ heart activities in real time to help determine the success of the model. A left intercostal thoracotomy was performed to partially expose the heart, and the left anterior descending coronary artery (LAD) was ligated with 8–0 nylon sutures for 30 min. After the ligation, we loosened the knot and reperfused the artery for 2 h. The sham group is a group that only opens the chest for threading without ligation.

The NPs were injected via tail vein five minutes before reoxygenation. The dose was 4.5 mg/kg. 

#### 2.2.12. Infarct size Determination

We injected Evans blue dye (2.0%) into the rat through its abdominal cardinal vein, and when the rat’s lips turned blue, the heart was rapidly excised and frozen at −26 °C for 15 min [31]. The hardened hearts were serially cut into 1 mm slices transversely and incubated in a pH 7.4, 1.0% 2, 3, 5 triphenyltetrazolium chloride (TTC) solution at 37 °C for 15 min. 

#### 2.2.13. Measurement of Left Ventricular Function

The rats were placed in a supine position. After the rats were reperfused, M-mode images of the left ventricle (LV; Visual Sonics Inc., Toronto, ON, Canada) were obtained from the parasternal short-axis view at the level of the papillar muscles and from the parasternal long-axis view [32], and the cardiac systolic function of the rats in different groups was compared. 

#### 2.2.14. Measurements of cTnT, MDA, LDH, SOD Activity and CK-MB 

Levels of cTnT, malondialdehyde (MDA), LDH, SOD activity, and CK-MB were measured using an assay kit according to the manufacturer’s protocols [33]. 

#### 2.2.15. Immunohistochemical Staining

For immunohistochemical (IHC) analysis, cardiac tissue sections (4 µm) were incubated with primary antibodies (1:200 dilution) for 12 h at 4 °C, followed by 1 h at room temperature with secondary antibodies. Sections were rinsed in PBS, and diaminobenzidine was added, followed by counterstaining with hematoxylin. Images were acquired using a pathology slide scanner [34]. We measured the area of positive staining with ImageJ software (National Institutes of Health, Bayesida, MD, USA). 

#### 2.2.16. Statistical Analysis

The data were displayed as the mean ± standard deviation. A Student’s *t*-test was utilized to evaluate data between the two groups, while the comparison among multiple groups was conducted by one-way ANOVA. Statistical analysis was performed with GraphPad Prism 6.0 software (GraphPad Software, San Diego, CA, USA). The significance level was set as *p* < 0.05. 

## 3. Results

### 3.1. SS31/MSN@PLTM Characterization

Table 1 presents the particle size and potential changes of SS31/MSN@PLTM within 24 h. The results showed that SS31/MSN@PLTM exhibited high stability without obvious aggregation. 

**Figure 2:**
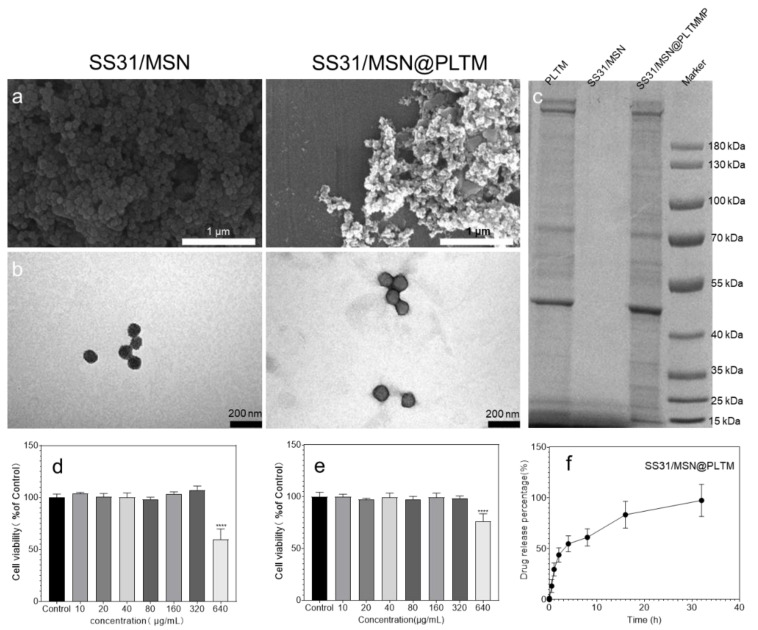
(**a**) SEM and (**b**) TEM images of SS31/MSN andSS31/MSN@PLTM; (**c**) SDS results of PLTM, SS31/MSN andSS31/MSN@PLTM; (**d**,**e**) the biosafety detection of SS31/MSN andSS31/MSN@PLTM (**** *p* < 0.001); and (**f**) the drug release curve of the material in PBS (pH 7.4).

The drug loading efficiency of SS31 was 52.3%. MSNs were purchased from Merck. The main properties of the MSNs were: diameter: 60–250 nm, specific surface area: 410–680 m^2^/g, pore size: 2.8–13.3 nm, pore volume: 0.57–1.66 cm^3^/g. To obtain the final SS31/MSN@PLTM, PLTM and SS31/MSN were wrapped by ultrasound [35]. To verify that MSNs were wrapped by PLTM successfully, we observed the differences in the appearance of the MSNs with and without PLTM (SS31/MSN@PLTM and SS31/MSN groups) by SEM and TEM [36]. As shown in Figure 2a,b, the PLTM was successfully wrapped on the outside of MSNs. Since the targeting function of PLTM was exerted by specific membrane proteins, we further performed SDS electrophoresis to verify that SS31/MSN@PLTM had the same protein expression as the PLTM. Figure 2c shows that SS31/MSN@PLTM and PLTM had consistent bands, further confirming that the PLTM was successfully wrapped. Figure 2d,e show the biocompatibility of SS31/MSN and SS31/MSN@PLTM. The presence or absence of the PLTM cloaking had no significant effect on cell viability, and these two materials were non-toxic to the cells within the concentration of 320 μg/mL. Figure 2f shows the drug release curve of the material in PBS with pH 7.4. SS31 was completely released over 16–32 h, and it could, therefore, play an anti-oxygen role during the reperfusion process. When MI/RI occurs, the ROS level surges in the first 5 min of reperfusion [4], and the drug release system can exert its antioxidant effect during the reperfusion process, allowing the drug to quickly block the vicious circle caused by the increase of ROS. 

### 3.2. Co-Localization and Cellular ROS Levels

**Figure 3:**
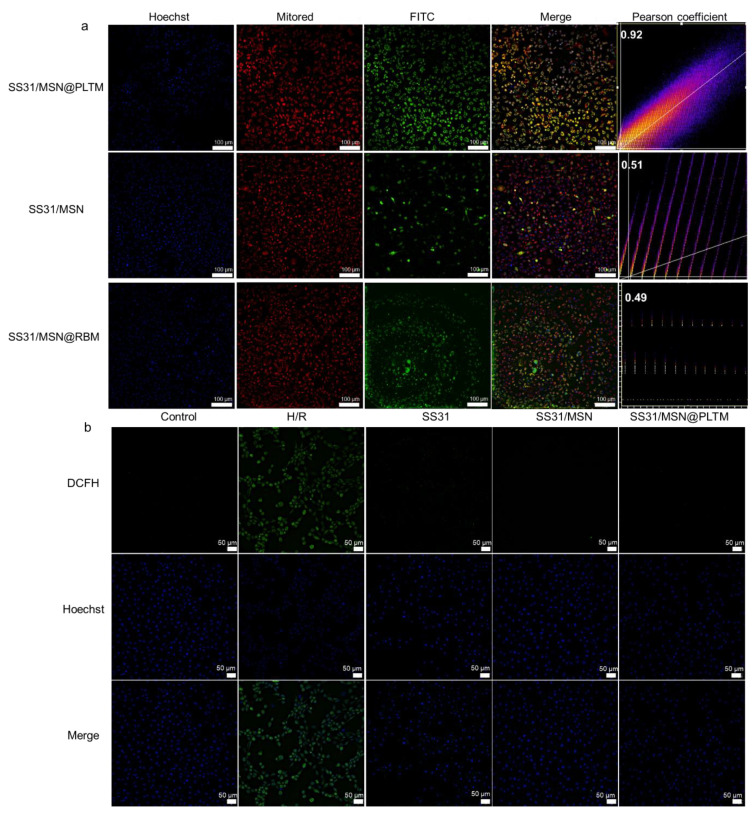
(**a**) Co-localization level of mitochondria and peptides (*n* = 3, scale bar is 100 μm) and (**b**) fluorescence intensity of cellular ROS level (*n* = 3, scale bar is 50 μm).

An H/R model was established to simulate ischemia-reperfusion injury. In myocardial ischemia, platelet membrane exhibits natural infarct-homing ability and can adhere to components on the exposed subendothelial ECM for drug targeting [19,20]. At the same time, SS31 has a mitochondrial targeting effect. Therefore, the targeting ability of the drug delivery system can be verified by detecting the co-localization level of the peptide and mitochondria. Based on this, we set the erythrocyte membrane-coated material SS331/MSN@RBM as a control, compared the co-localization level of FITC-labeled SS31 and Mito Red-labeled mitochondria in cells, and used the Pearson coefficient to quantify the degree of co-localization. The results are shown in Figure 3a. The Pearson coefficient of SS31/MSN@RBM and mitochondria was 0.49, which is similar to SS31/MSN, and far lower than 0.92 of SS31/MSN@PLTM group. This means that RBM does not have the effect of targeting damaged cells, indicating that PLTM could enhance the aggregation of SS31 in damaged cells. However, the results of co-incubating SS31/MSN@PLTM with RAW 264. 7 were completely different (Figure S1). The green fluorescence of the material did not appear in macrophages, indicating that SS31/MSN@PLTM was not phagocytosed by macrophages; thus, SS31/MSN@PLTM has stealth function. 

**Figure 4:**
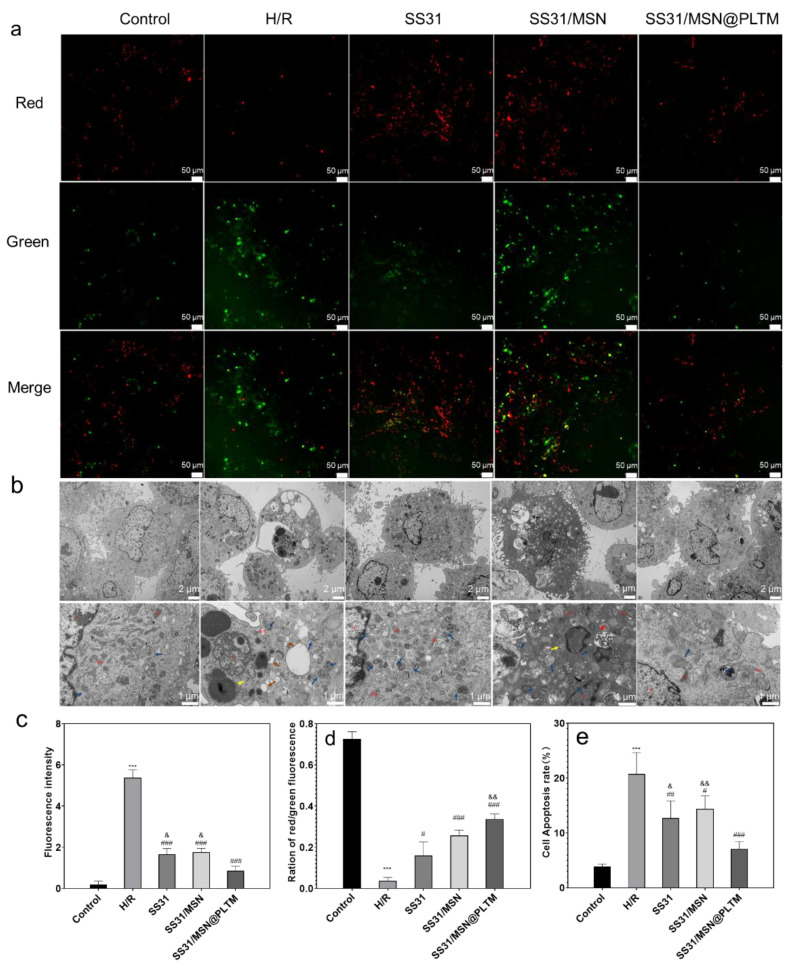
(**a**) Mitochondrial membrane potential (scale bar is 50 μm), (**b**) TEM images of cell apoptosis, Nucleus (N), mitochondria (Mi), rough endoplasmic reticulum (RER), Golgi apparatus (GB); microfilaments (↑), autophagy (↑), chromatin aggregation (↑), rough endoplasmic reticulum expansion cystic (↑), swollen mitochondria (↑), mild mitochondrial pyknosis (↑) (scale bars are 2 μm and 1 μm, respectively), and (**c**–**e**) statistical chart of ROS fluorescence level, JC-1 red–green ratio and cell apoptosis rate (*n* = 3; *** *p* < 0.001, compared with the control group; ^#^ *p* < 0.05, ^##^ *p* < 0.01, ^###^ *p* < 0.001, compared with H/R group; and ^&^ *p* < 0.05, ^&&^ *p* < 0.01, compared with SS31/MSN@PLTM group).

According to Figure 3b and Figure 4c, the three groups of drugs significantly reduced the increase of ROS content caused by H/R injury. Among them, SS31/MSN@PLTM exhibited the strongest antioxidant capacity, which was consistent with the co-localization results. In general, we can conclude that the PLTM can increase the biological function of the nanosystem on injured cardiomyocytes and this effect may be achieved through the adhesion function of the PLTM. 

### 3.3. SS31/MSN@PLTM Maintaining Mitochondrial Membrane Potential and Reducing Apoptosis

ROS can damage mitochondria and reduce mitochondrial membrane potential, which significantly leads to H/R damage. Whether SS31/MSN@PLTM can enhance the ability of SS31 to maintain mitochondrial membrane potential in H/R cells can be assessed by JC-1 [37]. As shown in Figure 4a,d, the SS31/MSN@PLTM group had the best ability to protect cells from the collapse of mitochondrial membrane potential. 

Mitochondrial damage can induce apoptosis. Therefore, cell apoptosis morphology was next observed by TEM, and the level of apoptosis in the H/R cells was assessed using flow cytometry [38]. As shown in Figure 4b, the cells in the control group had normal morphology. Significant autophagy, chromatin aggregation, and expansion of rough endoplasmic reticulum appeared in the cytoplasm of the cells in the H/R group. In addition, autophagy appeared in the cytoplasm of S group cells. Cells in the SM group exhibited apoptosis, with shrinking nuclei and swelling of mitochondria; cells in the SS31/MSN@PLTM group only had mild mitochondrial pyknosis and a small amount of autophagy. Meanwhile, as shown in Figure 4e, the SS31/MSN@PLTM group did better at reducing cell apoptosis caused by H/R. These results indicated that SS31/MSN@PLTM had the strongest protective effect on mitochondrial structure and the best anti-apoptotic effect. Again, SS31/MSN@PLTM seems to be an advantageous carrier for drug delivery.

### 3.4. SS31/MSN@PLTM Reducing Myocardial Infarct and Ischemic Sizes and Restoring Cardiac Function

**Figure 5:**
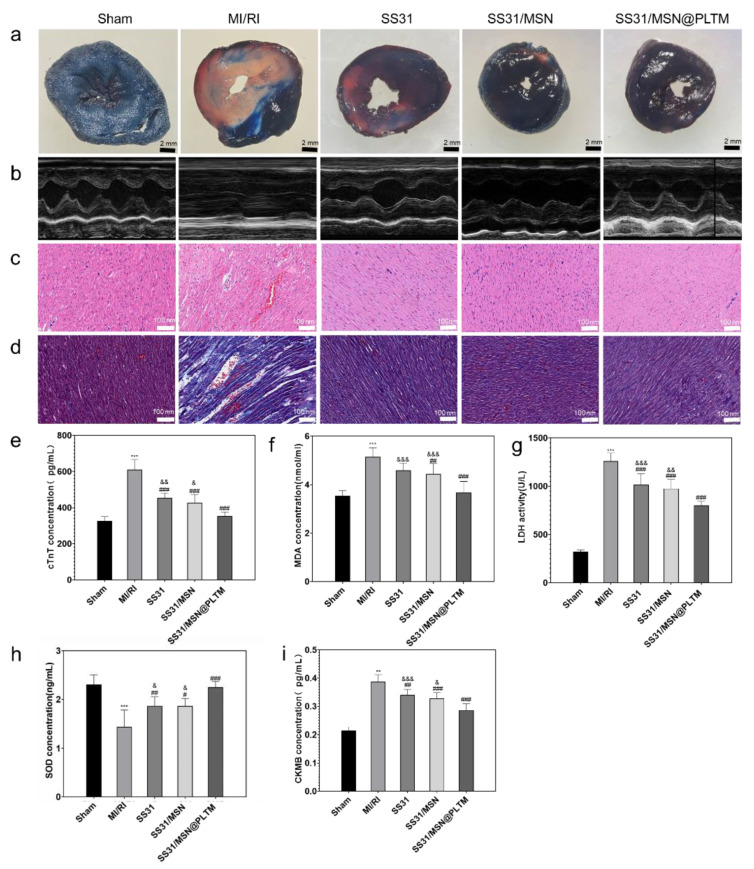
(**a**) Results of EB/TTC double staining (scale bar is 2 mm), (**b**) M-mode ultrasound, (**c**) HE staining (scale bar is 100 nm), (**d**) Masson staining (scale bar is 100 nm), and (**e**–**i**) the changes of cTnT, MDA, LDH, SOD, and CK-MB level of serum in different groups of rats (*n* = 3; *** *p* < 0.001, compared with sham group; ^###^ *p* < 0.001, ^##^ *p* < 0.01, ^#^ *p* < 0.05, compared MI/RI group; ^&&&^ *p* < 0.001, ^&&^ *p* < 0.01, ^&^ *p* < 0.05, compared with SS31/MSN@PLTM group).

To further evaluate the protective effect of SS31/MSN@PLTM on MI/RI, we established a MI/RI rat model [39]. The EB/TTC staining [24] of rat hearts is shown in Figure 5a. The infarct areas of rats in the S, SM, and SS31/MSN@PLTM groups are smaller than those in the MI/RI group, and the infarct area in SS31/MSN@PLTM group is the smallest. 

Cardiac function indicators can further show that SS31/MSN@PLTM has a higher protective effect on the heart than other treated groups [40,41]. Figure 5b shows that, compared with other groups, the heartbeat amplitude of the left ventricular long-axis section in the SS31/MSN@PLTM group was uniform, the ejection fraction was 65–70%, and no obvious arrhythmia was present.

### 3.5. SS31/MSN@PLTM Maintaining Myocardial Structure and Affecting the Levels of LDH, SOD, MDA, and CK-MB in Rat Serum

H&E and Masson staining (Figure 5c,d) showed that the myocardium in this group was consistent with the performance of the cardiac function. At the same time, the levels of myocardial injury markers LDH, cTnT, and CK-MB and the oxidation indexes MDA and SOD [9] in rat serum were detected (Figure 5e–i). The results showed that SS31/MSN@PLTM could enhance the effect of SS31, showing the best performance among the three treatment groups.

The above results demonstrate that loading SS31 into SS31/MSN@PLTM can enhance its cardiac protective function.

### 3.6. Effects of SS31/MSN@PLTM on the Expression of Apoptosis-Related Proteins

**Figure 6:**
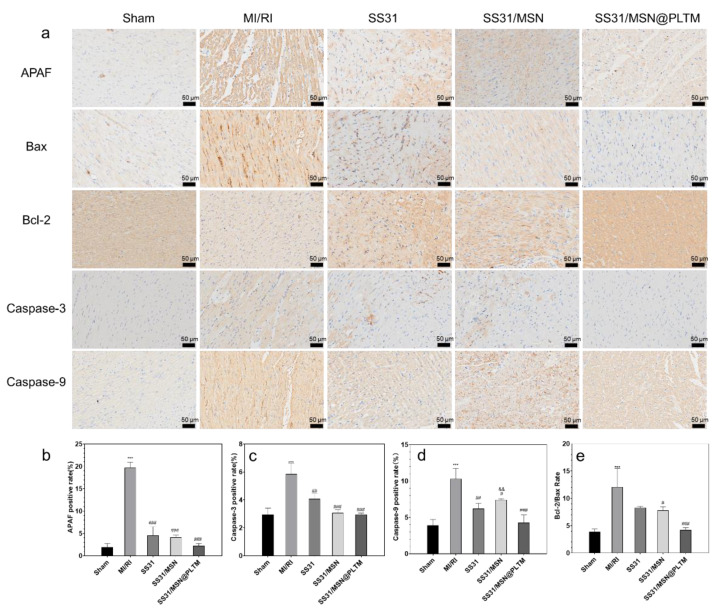
(**a**) Immunohistochemical staining of apoptosis-related proteins in rat myocardium (scale bar is 50 μm), (**b**) positive rate of APAF, (**c**) Caspase-3, (**d**) Caspase-9, and (**e**) Bcl-2-to-Bax ration (*n* = 3; *** *p* < 0.001, compared with Control group; ^###^ *p* < 0.001, ^##^ *p* < 0.01, ^#^ *p* < 0.05, compared with MI/RI group; ^&&^ *p* < 0.01, compared with SS31/MSN@PLTM group).

Elevated levels of ROS can damage cellular mitochondria, leading to apoptosis and affecting myocardial function. As an antioxidant, SS31 can inhibit apoptosis by maintaining mitochondrial structure stability through anti-ROS. To verify the anti-apoptosis effect of SS31, we selected apoptosis-related proteins APAF, Bcl-2, and Bax and apoptosis pathway-related proteins Caspase-3 and Caspase-9 for immunohistochemical staining (Figure 6) [42]. The results showed that, except for caspase-9, SS31/MSN@PLTM showed no significant difference compared with the other two groups. Bcl-2/Bax can indicate the opening degree of mPTP [43], and Figure 6e shows that no significant difference was present among the three groups. This result implies that the ischemia-reperfusion injury is not caused by ROS alone, but it also covers a broader complex of mechanisms such as mitochondrial damage induced apoptosis.

## 4. Discussion

In this study, we prepared a drug delivery system SS31/MSN@PLTM that can target the site of cardiovascular injury and release mitochondria-targeted antioxidant peptides SS31 at the site of injury. The drug delivery system was characterized, and the experimental results showed that the synthesized material had protein bands consistent with PLTM, and TEM results also showed that the PLTM was successfully encapsulated. The results of the biocompatibility experiment showed that the material had no obvious damage to cells within the range of 320 μg/mL, which means that the material was safe within this concentration range.

In the process of MI/RI, vascular damage and exposure of subendothelial matrix components, such as collagen and von Willebrand factor (vWF) can cause platelet aggregation to the injury site. At the same time, glycoproteins on the platelet surface, such as GPVI, GPIV, GPIb, GPIX, GPV, and GPIIb/IIIa, are also involved in platelet aggregation [44]. Therefore, the function of targeting the damaged cardiovascular site can be achieved through the encapsulation of the platelet membrane. In addition, since platelets are natural blood components and cannot be recognized by macrophages, they can achieve a stealth effect [45].

Since the peptide is easily degraded by proteases [46], loading SS31 into MSN for delivery can reduce the degradation of the peptide and improve the aggregation of the peptide at the damaged site. At the same time, SS31 can interact with cardiolipin independently of the mitochondrial membrane potential and specifically concentrate on the inner mitochondrial membrane. Therefore, the ability of the drug delivery system to target the damage site can be verified by detecting the degree of overlap between SS31 and the mitochondria. The results showed that SS31/MSN@PLTM can significantly increase the degree of overlap between peptides and mitochondria, which means that the coating of PLTM can improve the aggregation of SS31 at the damaged site.

In addition, the SS31 peptide can reduce the level of ROS [47,48,49], similar to SOD enzymes. This is because SS31 can selectively bind to cardiolipin on the inner mitochondrial membrane, and by protecting cardiolipin, it can effectively inhibit the production of intracellular ROS under pathological conditions and relieve oxidative stress [50]. The results showed that SS31/MSN@PLTM can significantly reduce the increase of ROS level caused by hypoxia and reoxygenation in cells, and maintain the stability of mitochondrial membrane potential.

Mitochondrial dysfunction can lead to cardiomyocyte apoptosis [43], and observation of cell ultrastructure shows that SS31/MSN@PLTM can reduce cell apoptosis caused by hypoxia and reoxygenation.

Cardiac infarct size and cardiac function are important indicators for judging the degree of injury. The results showed that SS31/MSN@PLTM can reduce myocardial infarct size, restore cardiac systolic function and myocardial structure, and reduce the level of myocardial injury markers. The mitochondrial apoptosis pathway caused by ischemia-reperfusion triggers a series of cascade reactions. Bcl-2 and Bax proteins are key factors in regulating the opening of mitochondrial permeability transition pore (mPTP) [51,52]. It causes the release of cytochrome C, which can bind to Apaf-1 and activate Caspase-9, which in turn activates Caspase-3 to induce apoptosis of cardiomyocytes [53,54]. The apoptosis-inducing factor (AIF) plays a key role in the caspase family-independent apoptosis process [55]. It is also released from mitochondria to the cytoplasm after mPTP is opened. The experimental results show that SS31/MSN@PLTM can reduce mPTP pores opening. The expression of apoptosis-related proteins ultimately improved cardiac function in rats.

## 5. Conclusions

This study successfully synthesized a PLTM encapsulated enzyme–mimicking drug release system, which could effectively target injured cardiovascular cells, release drugs at specific injury sites, reduce the apoptosis caused by MI/RI, improve myocardial function, and reduce the expression level of cardiomyocyte apoptosis-related proteins. The drug delivery system may be a potential drug for the clinical treatment of MI/RI.

## Figures and Tables

**Table 1:** Particle size and potential of SS31/MSN@PLTM at different time points (*n* = 3, ±SD).

	0 h	1 h	3 h	6 h	12 h	24 h
Particle size(nm)	80.84 ± 3.17	84.75 ± 4.60	83.56 ± 3.39	83.55 ± 3.10	86.43 ± 3.67	84.79 ± 3.64
Zeta potential (mV)	−1.35 ± 0.12	−1.26 ± 0.17	−1.1 ± 0.25	−1.14 ± 0.40	−1.06 ± 0.32	−1.23 ± 0.29

## Data Availability

Not applicable.

## Supplementary Materials

The following supporting information can be downloaded at: https://www.mdpi.com/article/10.3390/jfb13040181/s1, Figure S1: Fluorescence images of RAW 264.7 cells with SS31/MSN@PLTM.
