# Supplementary material for: Platelet Membrane–Encapsulated MSNs Loaded with SS31 Peptide Alleviate Myocardial Ischemia-Reperfusion Injury
Source: J Funct Biomater. 2022 Oct 9;13(4):181. doi: 10.3390/jfb13040181 (PMC9624354; doi:10.3390/jfb13040181)
Supplement: Supplementary file 1 [file jfb-13-00181-s001.zip › jfb-1884572-supplementary.pdf]

Additional informaition for:

Platelet membrane–encapsulated MSNs loaded with SS31 peptide alleviate myocardial ischemia-reperfusion injury

Zaiyuan Zhang [a,1], Zhong Chen [b,1], Ling Yang [c,1], Jian Zhang [a], Yubo Li [d], Chengming Li [c], Rui Wang [b], Xue Wang [c], Shuo Huang [c], Yonghe Hu [a**], Jianyou Shi [e**], Wenjing Xiao [f,g *]

The file includes: **Figure S1**

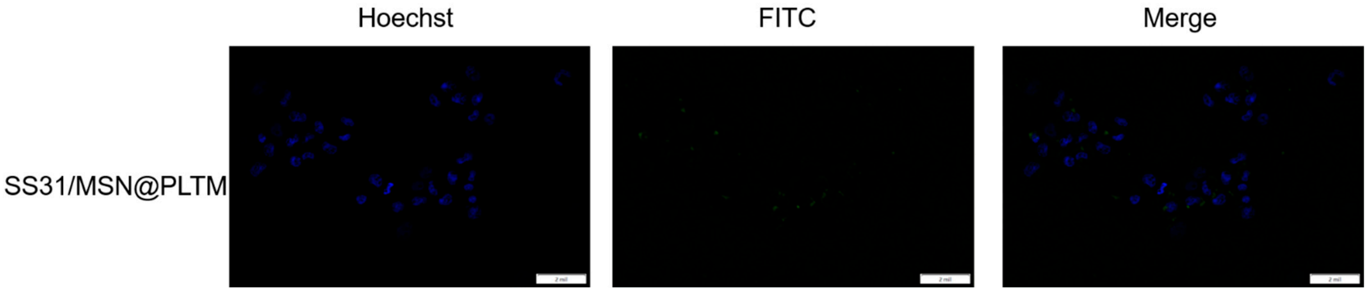

Figure S1. Fluorescence images of RAW 264.7 cells with SS31/MSN@PLTM.
